# The complete chloroplast genome of the moss, *Myurella julacea* (Schwägr.) Schimp. (Bryidae, Pterigynandraceae)

**DOI:** 10.1080/23802359.2020.1825138

**Published:** 2020-10-07

**Authors:** Yeong-Deok Han, SeungJin Park, Young-Jun Yoon

**Affiliations:** a Research Center for Endangered Species, National Institute of Ecology, Yeongyang-gun, The Republic of Korea; b Department of Biology, Jeonbuk National University, Jeonju, The Republic of Korea

**Keywords:** *Myurella julacea*, complete chloroplast genome, moss

## Abstract

Here, we report the complete chloroplast (cp) genome of the moss *Myurella julacea* (Schwägr.) Schimp. We found that the total length of the *M. julacea* complete cp genome was 124,457 base pairs (bp) long, comprising 82 protein-coding genes, 36 tRNA genes, and 8 rRNA genes. The genome had a typical quadripartite structure, and consisted of a large single-copy region (LSC) of 86,607 bp, a small single-copy region (SSC) of 18,508 bp, and a pair of inverted repeats with a length of 9671 bp each. The base composition of the cp DNA was 26.0% A, 29.4% T, 24.5% C, and 20.1% G with an overall GC content of 44.6%. Phylogenetic analysis revealed that *M. julacea* clustered into a clade with other Hypnales groups with high bootstrap support. The complete cp genome presented here will provide useful information for phylogenetic and evolutionary studies of endangered Bryophyte species.

The genus *Myurella* was established by Schimp. in Bryologia Europaea for one species, *Myurella julacea* (Schwägr.) Schimp. The small mouse-tail moss (*M. julacea*) is a species of the family Theliaceae found in the mountainous areas of Japan, China, Siberia, Central Asia, Kashmir, Caucasus, Europe, and North America (Noguchi 1991). This minute moss has shoots that are only about 1–3 cm long and less than 0.5 mm wide. Fresh shoots are smoothly cylindrical, wiry branched, and silvery blue-green in color. It grows in rock crevices at calcareous sites (Noguchi 1991). *Myurella julacea* can be distinguished from allied species in South Korea by its strongly julaceous branches, rounded ovate leaves, and axillary gemmae (Noguchi 1991).

We collected fresh samples from Jeongseon-gun (37°27′05.9″N; 128°41′05.0″E), Gangwon-do, South Korea and stored them at the Jeonbuk National University in Jeonju, Korea, with the accession number PSJ-17123151. Genomic DNA extraction was performed using the DNeasy^®^ Plant Mini Kit (Qiagen, Hilden, Germany) according to the manufacturer’s instructions. The DNA library was constructed using a QIAseq FX Single Cell DNA Library Kit (Qiagen, Germany), and the library was sequenced using paired-end (2 × 150 bp) sequencing using the Illumina HiSeq platform (Illumina, San Diego, CA). The cp genome was assembled by Program Geneious 8.1.9 using the partial sequences of the ribosomal protein S4 (*rps4*) of *M*. *julacea* (GenBank accession no. MG199031) as the seed sequence. Gene annotation was performed using Geneious 8.1.9 as the base cp genome of *Climacium dendroides* (GenBank accession no. MT006132), and then manually modified compared to the complete cp genomes of other members of the Bryopsida class (Han et al. 2020).

The cp genome of *M. julacea* (MT809490) had a total length of 124,457 bp and consisted of 82 protein-coding genes (PCGs), 36 tRNAs, and 8 rRNAs, which were similar to the gene arrangement of other Bryophyta cp genomes. The cp genome of this organism had a typical quadripartite structure consisting of large and small single-copy regions (LSC 86,607 bp and SSC 18,508 bp) separated by a pair of 10,014 bp-long inverted repeat (IR) regions. The nucleotide composition consisted of 35.5% A, 14.5% C, 14.4% G, and 35.6% T. The overall AT content was 71.1%, and the AT contents of the LSC, SSC, and IR regions were 74.0%, 74.3%, and 55.4%, respectively.

The dataset used for the construction of the phylogenetic tree consisted of the concatenated alignment sequences of 16 PCGs obtained from the complete cp genomes of 26 Bryophyta, and two Marchantiophyta according to Cevallos et al. (2019). A maximum likelihood (ML) tree was inferred based on the JTT matrix-based model using MEGA X, with 1000 bootstrap replicates. In conclusion, the analysis of *M. julacea* datasets provides convincing evidence that supports many traditionally recognized families and contributes to the understanding of the structure of the complete cp genome (Figure 1) (Cox et al. 2000; Magombo 2003; Liu et al. 2014). Additionally, the cp genome sequence of *M. julacea* offers a useful resource for future genetic and phylogenetic conservation studies.

**Figure 1.**
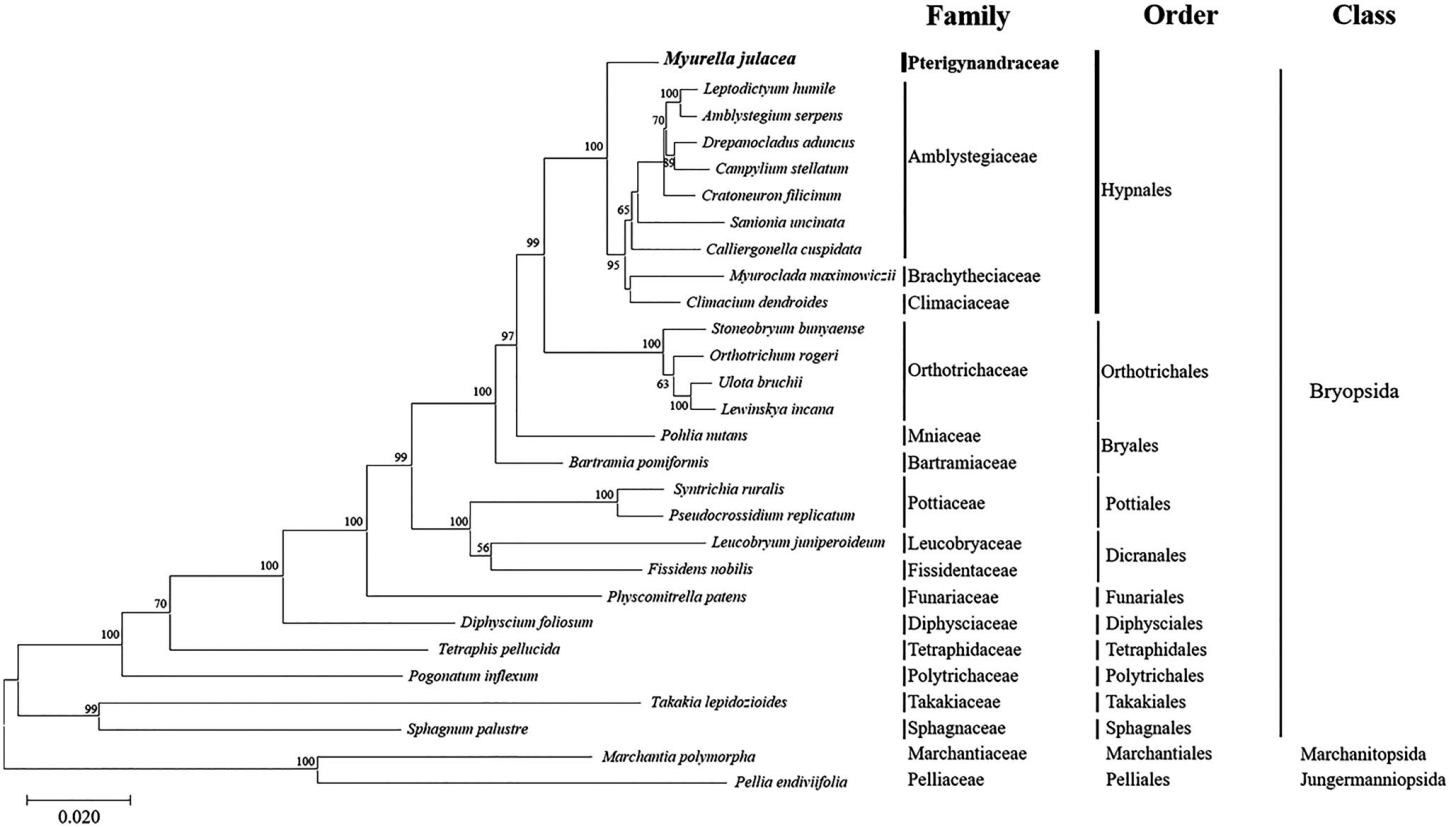
Phylogenetic position of *M*. *julacea* (MT809490) determined by maximum likelihood methods based on combined analysis with amino acids sequences of 28 complete chloroplast genome common in all taxa. Bootstrap values over 50% from 1000 replicates are exhibited for corresponding branches. *Marchantia polymorpha* (GenBank accession no. NC_037507) and *Pellia endiviifolia* (NC_019628) are used as outgroup. GenBank accession numbers of complete chloroplast genome used are *Amblystegium serpens* (NC_049069), *Bartramia pomiformis* (MT024676), *Calliergonella cuspidata* (NC_049070), *Campylium stellatum* (NC_049072), *Climacium dendroides* (MT006132), *Cratoneuron filicinum* (NC_049073), *Diphyscium foliosum* (NC_046057), *Drepanocladus aduncus* (NC_049074), *Fissidens nobilis* (NC_044155), *Leptodictyum humile* (NC_049075), *Leucobryum juniperoideum* (MK952779), *Lewinskya incana* (NC_042174), *Myuroclada maximowiczii* (MT726030), *Orthotrichum rogeri* (NC_026212), *Physcomitrella patens* (NC_037465), *Pogonatum inflexum* (MK131349), *Pohlia nutans* (NC_045869), *Pseudocrossidium replicatum* (MG132071), *Sanionia uncinata* (NC_025668), *Sphagnum palustre* (NC_030198), *Stoneobryum bunyaense* (NC_049071), *Syntrichia ruralis* (FJ546412), *Takakia lepidozioides* (NC_028738), *Tetraphis pellucida* (NC_024291), and *Ulota bruchii* (NC_042480).

## Data Availability

The data that support the findings of this study are openly available in GenBank of NCBI at https://www.ncbi.nlm.nih.gov, reference number MT726030.
